# Bluetooth Mesh Energy Consumption: A Model

**DOI:** 10.3390/s19051238

**Published:** 2019-03-12

**Authors:** Seyed Mahdi Darroudi, Raül Caldera-Sànchez, Carles Gomez

**Affiliations:** Department of Network Engineering, Universitat Politècnica de Catalunya/Fundació i2Cat, C/Esteve Terradas, 7, 08860 Castelldefels, Spain; sm.darroudi@entel.upc.edu (S.M.D.); raulcs1995@gmail.com (R.C.-S.)

**Keywords:** Bluetooth Mesh, Bluetooth Low Energy, BLE, energy, modeling, performance, evaluation, Internet of Things, IoT

## Abstract

The recent publication of the Bluetooth Mesh standard is a remarkable milestone in the evolution of Bluetooth Low Energy (BLE). As a new technology in the Internet of Things (IoT) market, it is crucial to investigate the performance of Bluetooth Mesh. However, while a fundamental feature of Bluetooth Mesh is its suitability for energy-constrained devices, this aspect has not yet been properly considered in the literature. In this paper, we model the current consumption, lifetime and energy cost per delivered bit of a battery-operated Bluetooth Mesh sensor node. The model is based on measurements performed on a real hardware platform. Evaluation results quantify the impact of crucial Bluetooth Mesh parameters. Among others, we have found that a sensor device running on a simple 235 mAh battery, and sending a data message every 10 s, can achieve a lifetime of up to 15.6 months, whereas the asymptotic lifetime is 21.4 months.

## 1. Introduction

Bluetooth Low Energy (BLE) is a low-power wireless technology that has become a cornerstone for the Internet of Things (IoT) [1,2]. With a privileged presence in massively used consumer electronics devices, BLE has enormous potential to enable interaction between a user and surrounding IoT devices. 

BLE was originally designed to create star-topology networks, where devices with significant resource constraints are connected via a direct link to a potentially less constrained central device. An example of this paradigm is a set of battery-operated sensors that provide their readings to a smartphone that centralizes sensor data collection. However, the star network topology intrinsically limits the geographical area covered by a BLE network. In addition, BLE links are typically of a rather short range, in the order of few tens of meters, which further reduces the applicability of BLE for relevant IoT applications. In contrast, many competing IoT technologies support the mesh topology, which allows extended network coverage and also provides robustness by means of path diversity [3].

In order to overcome the limitations of the star-topology-based BLE design, significant efforts have been devoted by industry, academia and standardization bodies to create BLE mesh networks [4]. Remarkably, the Bluetooth SIG recently published the Bluetooth Mesh suite of specifications, which offers a standardized solution for end-to-end communication in a mesh network composed of BLE devices [5,6,7]. As a relevant new connectivity solution in the IoT market, it is crucial to investigate the performance of Bluetooth Mesh. Despite the novelty of this technology, there are already several published research studies on Bluetooth Mesh [8,9,10,11,12,13,14]. Existing work focuses on performance parameters such as Packet Delivery Ratio (PDR), latency and throughput. However, while a fundamental feature of Bluetooth Mesh is its suitability for energy-constrained devices, this aspect has not yet been properly considered in the literature.

In this paper, we model the energy performance of a low power device (more specifically, a battery-operated sensor) that makes use of the energy saving feature provided by Bluetooth Mesh. We call such a device a Bluetooth Mesh-Low Power Node (BM-LPN). We develop analytical models for three important energy performance parameters: the BM-LPN average current consumption, the theoretical lifetime of a battery-operated BM-LPN, and the energy consumed per each user data bit delivered by the BM-LPN. Our models, which are derived from measurements performed on real hardware, allow us to quantify the impact of critical Bluetooth Mesh parameters, the BM-LPN data message rate, and the frame loss rate, on BM-LPN energy performance. We have found that a BM-LPN running on a simple 235 mAh battery, and sending a data message every 10 s, can achieve a lifetime of up to 15.6 months with the considered hardware platform, whereas the asymptotic lifetime is 21.4 months. We also discuss the applicability and limitations of Bluetooth Mesh as an IoT technology, considering the power supply requirements that stem from the Bluetooth Mesh architectural design.

The remainder of this paper is organized as follows. Section 2 analyzes the literature related to this work. Section 3 gives an overview of Bluetooth Mesh and its low power support features. Section 4 presents the BM-LPN current consumption, lifetime and per-delivered-bit energy consumption models. Evaluation results are provided and discussed in Section 5. Section 6 reflects on the Bluetooth Mesh architecture and its applicability as an IoT technology. Finally, Section 7 concludes the paper with the main remarks. 

## 2. Related Work

BLE mesh networking design, evaluation and improvement has become a topic of interest for the community in the last years [4]. The publication of the Bluetooth Mesh suite of specifications [5,6,7] in July 2017 was a landmark event that offered a standardized solution for BLE mesh networking. (Note that, as of this publication, another specification is being developed at the Internet Engineering Task Force (IETF) in order to support IPv6 over mesh networks of BLE devices, which however follows a different approach from that of Bluetooth Mesh [15].) Despite its novelty, Bluetooth Mesh has already been the subject of several research works, which we review next. 

Baert et al. provide an overview on Bluetooth Mesh and a thorough investigation of the Round Trip Time (RTT) in this technology, which is carried out both analytically and also experimentally [8]. However, their work does not consider energy-related performance parameters. Veiga and Abbas propose a Bluetooth Mesh profile for smart cities, and implement a proof-of-concept system. However, they do not carry out any performance evaluation [9]. Wan and Liu create a smart-home architecture based on Bluetooth Mesh. While they describe the fundamentals of their design, and they validate it by using a 14-node testbed, they do not evaluate network performance [10]. Deknache and Nilsson conduct an indoor and outdoor experimental evaluation of Bluetooth Mesh, focusing only latency and throughput [11]. Söderholm adds routing functionality based on Bloom filters on top of a simulated Bluetooth Mesh network, which improves PDR; however, energy performance is not considered in the work [12]. Berglund evaluates Bluetooth Mesh in a real industrial environment, focusing only on PDR as a performance parameter [13]. 

The only published work that, to our best knowledge, considers energy consumption as an evaluation parameter in the context of Bluetooth Mesh to some extent is authored by Martínez et al. [14]. They develop a smart doorbell proof-of-concept implementation on a 4-node real testbed. While they focus mainly on PDR as the main performance parameter in their study, they also measure the current consumption of an nRF52 module during sleep mode and estimate a maximum device lifetime of 50 days for a battery capacity of 240 mAh. However, they do not measure or model the device current consumption in any other state, and do not consider the specific Bluetooth Mesh mechanisms for low power operation.

## 3. Bluetooth Mesh and Low Power Support

This section first provides an overview of Bluetooth Mesh. Secondly, the section focuses on the features that enable low power support for energy-constrained devices.

### 3.1. Bluetooth Mesh Overview

**Figure 1:**
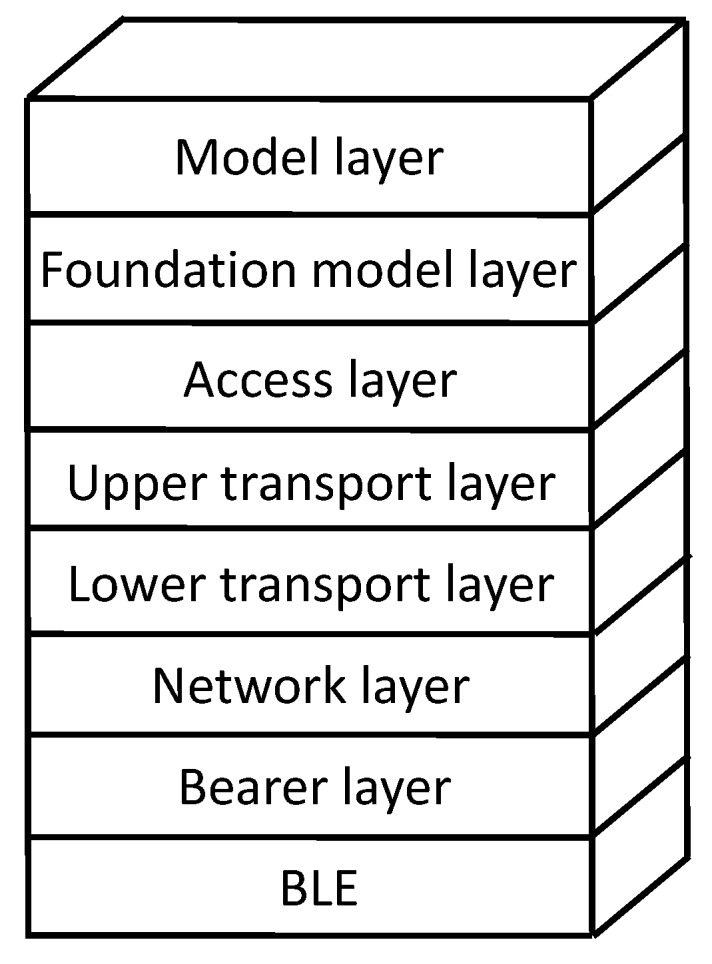
Bluetooth Mesh protocol stack.

The Bluetooth Mesh standard consists of a full protocol stack (Figure 1). At the lowest layer, BLE is leveraged as the means for the physical transmission of Bluetooth Mesh messages. On top of BLE, Bluetooth Mesh defines a set of layers that provide networking and application support functionality. We next review BLE, as well as the protocol layers introduced by the Bluetooth Mesh specifications.

#### 3.1.1. BLE Overview

BLE is defined by the Bluetooth core specifications as a layered protocol stack [1]. In the context of this paper, the two lowest layers of BLE (i.e., the Physical Layer and the Link Layer) are the most relevant ones. The Physical Layer defines 40 channels in the 2.4 GHz band, which are organized into 3 advertising channels and 37 data channels. The Physical Layer bit rate in Bluetooth 4.x is 1 Mbit/s, whereas further bit rate options are allowed in Bluetooth 5 [16]. 

In BLE, there exist two approaches for communication between neighboring nodes. The most simple one is based on broadcasting data by means of advertising channels. Messages sent over advertising channels are called advertisements. Typically, transmitting an advertisement leads to sending a copy of the advertisement over each one of the 3 advertising channels. The second approach for BLE communication is based on the establishment of a Link Layer connection between two neighbors, which provides bidirectional data exchange over data channels. 

#### 3.1.2. Bluetooth Mesh Layers

This subsection offers a brief layerwise, bottom-up, introduction to the Bluetooth Mesh protocol stack layers above BLE. These comprise the Bearer layer, the Network layer, the Lower Transport layer, the Upper Transport layer, the Access layer, the Foundation Model layer, and the Model layer. The Bearer layer defines the lower layer bearer to be used for message transmission. Typically, Bluetooth Mesh messages are sent as advertisements. The Network layer delivers end-to-end data units by means of a controlled flooding mechanism. The Lower transport layer offers reliable segmentation and reassembly for large data units. The Upper transport layer provides support for BM-LPNs, based on a concept called friendship (see Section 3.2), as well as end-to-end security. The Access layer provides optional end-to-end reliability. The Foundation model layer offers support for management and configuration of a Bluetooth Mesh network. Finally, the Model layer defines a framework for applications.

#### 3.1.3. Bluetooth Mesh Network Architecture 

Bluetooth Mesh enables end-to-end communication over a mesh network topology. To this end, Bluetooth Mesh defines the architectural elements called Bluetooth Mesh-Relay Nodes (BM-RNs). BM-RNs are the core components of a Bluetooth Mesh network. They are in charge of receiving and forwarding data messages, by means of a controlled flooding mechanism. They constitute a backbone to which nodes without relaying functionality support can connect to. A special type of BM-RNs is Bluetooth Mesh-Friend Nodes (BM-FNs), which offer connectivity support for BM-LPNs (see Section 3.2).

**Figure 2:**
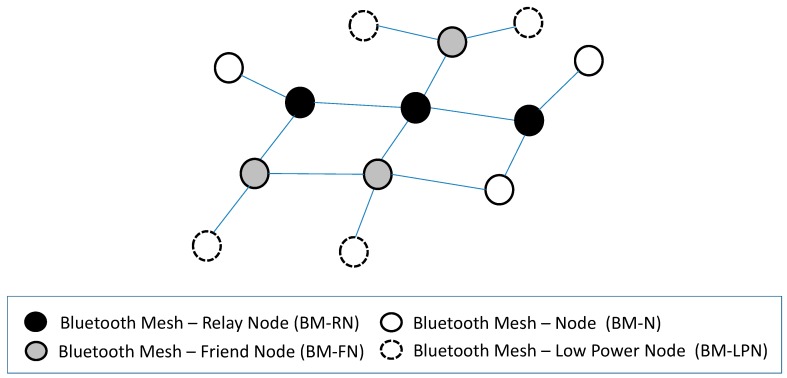
Bluetooth Mesh network topology and device roles.

Figure 2 illustrates an example Bluetooth Mesh network. Bluetooth Mesh-Nodes (BM-Ns) correspond to power-affluent devices that can participate in a Bluetooth Mesh network as a source or destination, but not as a relay.

### 3.2. Low Power Support in Bluetooth Mesh

**Figure 3:**
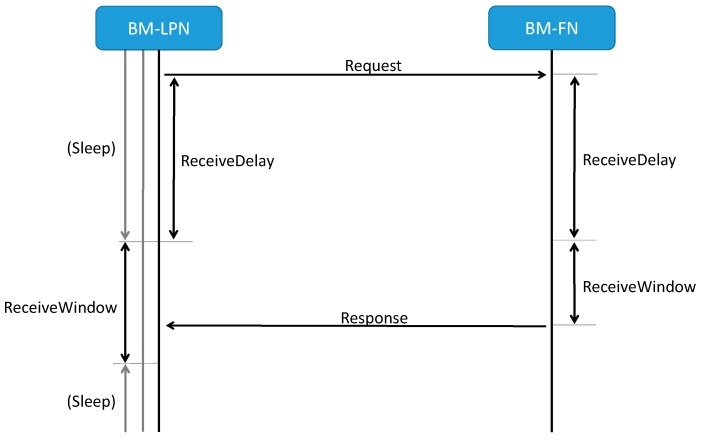
Illustration of a BM-LPN polling a BM-FN, and the related Bluetooth Mesh parameters involved.

Since BM-LPNs run on limited energy sources (e.g., small batteries), they remain by default in sleep mode in order to save energy. BM-LPNs can transmit messages at any time, since it is assumed that at least one of their next hop devices will be always ready to receive and forward such messages. However, in order to allow BM-LPNs be able to also receive messages, Bluetooth Mesh Upper transport layer defines the concept of a friendship, which is a special relationship between a BM-LPN and a one-hop neighbor which we refer to as Bluetooth Mesh-Friend Node (BM-FN). The latter, which is selected by the former among its one-hop neighbors, stores messages intended for the BM-LPN while this node is in sleep state. The BM-LPN asynchronously polls the BM-FN for possible incoming messages by sending a request message to the latter. After sending the request, the BM-LPN returns to sleep mode. After ReceiveDelay ms, which allow the BM-FN to prepare a response, the BM-LPN starts listening for up to ReceiveWindow ms. Upon receipt of a request, the BM-FN can send a stored message to the BM-LPN, if any. After receiving the last stored message, or after the end of the receive interval, the BM-LPN enters sleep mode again. The maximum time between two consecutive requests is defined by the PollTimeout parameter. Figure 3 depicts a diagram that represents the described polling process.

**Figure 4:**
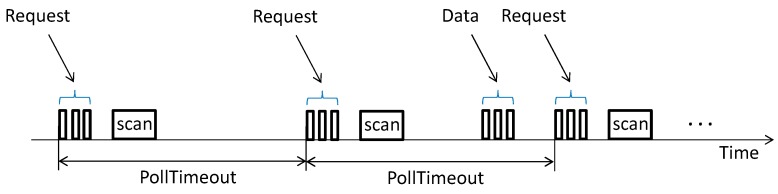
Polling and data message transmission operations carried out by a BM-LPN. In this example, data message transmission is only performed in the second PollTimeout interval.

Typically, a BM-LPN running as a sensor device will periodically poll the BM-FN and scan the channel after each request for ReceiveWindow ms, in addition to sending data messages containing sensor readings (see Figure 4). Note that, since advertisements are generally used to carry Bluetooth Mesh messages, one request or one data message transmission is usually performed by sending 3 advertisements. 

## 4. Modeling the Energy Consumption of a Bluetooth Mesh Low Power Node

In this section we present analytical models of three important energy consumption parameters of a battery-enabled BM-LPN: average current, battery lifetime, and energy consumed per bit delivered to a neighbor. We assume a BM-LPN running as a battery-operated sensor node that periodically transmits a data message (e.g., containing a sensor reading).

Our first goal is determining the BM-LPN average current consumption, denoted *I_average_*. As introduced in Section 3.2, the BM-LPN stays in sleep mode by default, and every PollTimeout sends a request to its BM-FN. In addition, the BM-LPN transmits a data message once every *T_Data_*. 

In order to capture realistic behavior, we derive our BM-LPN current consumption model based on measurements carried out on a real BLE device. However, probably due to the novelty of Bluetooth Mesh, the friendship feature is not currently supported in available Bluetooth Mesh implementations. For this reason, we create a model based on the three main actions performed by a BM-LPN when it is not in sleep mode: (i) transmitting three advertisements; and (ii) scanning the channel. The first action allows modeling the transmission of a request by the BM-LPN, as well as a data message transmission. The second one is useful to model the channel listening performed by the BM-LPN to check for potentially incoming data after a request. We identify and characterize the duration and current consumption of all relevant states corresponding to these two actions, separately, as measured on the tested device. 

**Figure 5:**
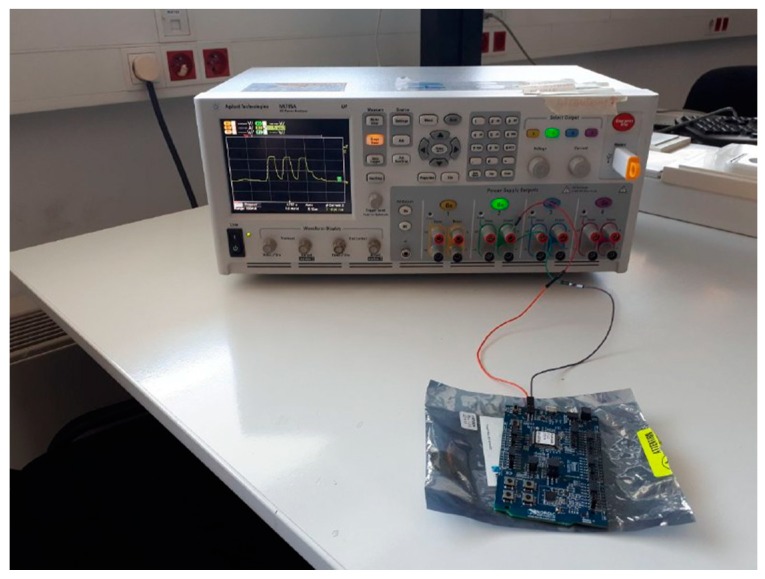
Experimental setup for the current consumption characterization of the nRF51422 PCA10028 board (front) using an Agilent N6705A power analyzer (back).

The device model used in our measurements is a PCA10028 Development Kit. This board includes an nRF51422 chipset, which belongs to the popular nRF51 series (Nordic Semiconductor, Trondheim, Norway) [17]. This module supports Bluetooth 4.2, as well as a Bluetooth Mesh implementation provided by the manufacturer [18]. During the measurements, the device transmit power is set to 4 dBm. Results are obtained by using an Agilent N6705A power analyzer (Agilent Technologies, Santa Clara, CA, USA). Figure 5 illustrates the experimental environment.

**Figure 6:**
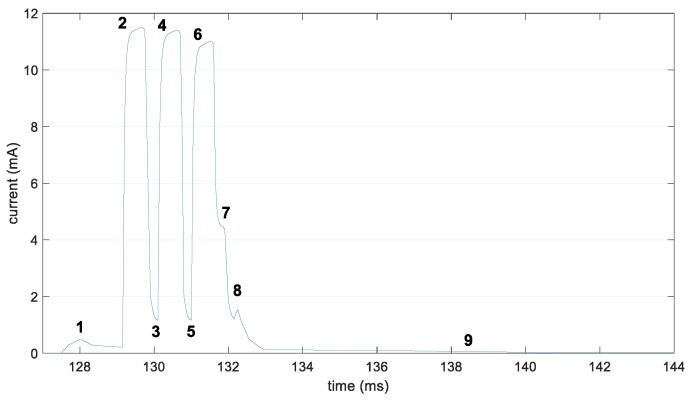
Current consumption of an nRF51422 Development Kit for the different states related with the transmission of three advertisements.

The current consumption profile of the device during the transmission of three advertisements is illustrated in Figure 6. Table 1 shows the related states, as well as their current consumption and duration values, along with their corresponding variables used in this paper. The values shown in Table 1 are obtained as the average from 10 individual experiments. Initially, the device wakes up from sleep mode (state 1), in order to prepare for the transmission of the advertisements. Then, the device transmits a first advertisement (state 2), followed by an interval during which the frequency channel of the radio is changed (state 3). Using the new channel, a second advertisement transmission is performed (state 4), followed by a second frequency channel change (state 5). Then, the third advertisement transmission is carried out (state 6), followed by an interval where the device turns off the radio (state 7), and a post-processing interval follows (state 8). Then, the device prepares for returning to the sleep state over a cool-down interval (state 9). Note that, in our model, we assume that the BM-LPN will return to the sleep state before scanning for incoming messages from the Friend Node in order to save energy, since the ReceiveDelay parameter may take values between 10 ms and 511 ms.

**Figure 7:**
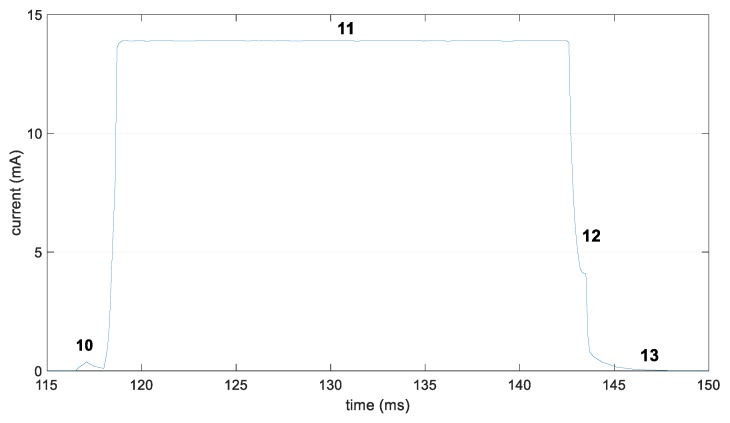
Current consumption of an nRF51422 Development Kit for the states related with performing a scan interval.

We next provide the device current consumption profile that corresponds to the scanning action (Figure 7 and Table 2). After the initial sleep state, the device wakes up (state 10) and sets the radio interface in receive mode during the whole scan interval (state 11). Subsequently, the device turns off the radio interface (state 12) and performs a cool-down sequence (state 13) in preparation for returning to sleep mode.

Based on the profiles of the advertisement transmissions and the scanning interval, we next calculate the BM-LPN average current consumption. Since the latter performs a request-scan cycle every PollTimeout, and it transmits a sensor reading every *T_Data_*, *I_average_* can be obtained as shown next:

(1) $$I_{average}=\frac{1}{\mathrm{PollTimeout}}\left(\overset{13}{\underset{i=0}{\sum }}T_{i}\cdot I_{i}+\frac{\mathrm{PollTimeout}}{T_{Data}}\overset{9}{\underset{i=1}{\sum }}T_{i}\cdot I_{i}\right)$$

where *I_i_* and *T_i_* denote respectively the current consumption and the duration of state *i* in Table 1 and Table 2. 

We next determine the average duration of the sleep interval within a PollTimeout interval, *T_sleep_*. Let *T_act_* be the average total duration of all states wherein the device is not in sleep mode. Then, *T_sleep_* can be calculated as:

(2) $$T_{sleep}=\mathrm{PollTimeout}-T_{act}$$

where *T_act_* can be obtained as shown in the next equation:

(3) $$T_{act}=\overset{13}{\underset{i=1}{\sum }}T_{i}+\frac{\mathrm{PollTimeout}}{T_{Data}}\overset{9}{\underset{i=1}{\sum }}T_{i}$$

As can be seen, *I_average_* can be determined by using Equations (1)–(3). Based on this performance parameter, it is possible to obtain the theoretical lifetime of a BM-LPN, denoted *T_lifetime_*, assuming a battery capacity of *C_battery_* (expressed in mA·h), and a battery self-discharge current, *I_self-discharge_*, as shown next:

(4) $$T_{lifetime}=\frac{C_{battery}}{I_{average}+I_{self-discharge}}$$

Note that the previous equation aims at capturing an important aspect of a realistic battery behavior, which is the degradation of its characteristics over time. We assume that *I_self-discharge_* is a constant value as a function of time.

Finally, we also model the energy consumed by the BM-LPN per user data bit delivered to a neighbor, *EC_delivery_*. Let *V* indicate the battery voltage of the BM-LPN. Let *E*[*l_delivery_*] denote the expected number of user data bits delivered per *T_Data_*. We obtain *EC_delivery_* as shown next:

(5) $$EC_{delivery}=\frac{I_{average}\cdot V\cdot T_{Data}}{E\left[l_{delivery}\right]}$$

where *E*[*l_delivery_*] depends on the Frame Loss Rate (FLR), and on the payload size, denoted *l_Payload_*. The data message sent by the BM-LPN will be correctly delivered to a next hop if at least one of the corresponding three individual advertisement transmissions that carry the data message is successfully received. Then, assuming that frame losses are uncorrelated, the expected amount of data delivered by the device per transaction is determined as:

(6) $$E\left[l_{delivery}\right]=l_{Payload}\cdot \left(1-{\mathrm{FLR}}^{3}\right)$$

## 5. Evaluation

In this section, we evaluate current consumption, lifetime, and the energy consumed per delivered bit of a battery-operated BM-LPN, by using the models provided in Section 4. This section is organized into three different subsections. Each subsection provides evaluation results, along with the corresponding discussion, for each aforementioned energy performance parameter.

### 5.1. BM-LPN Current Consumption

**Figure 8:**
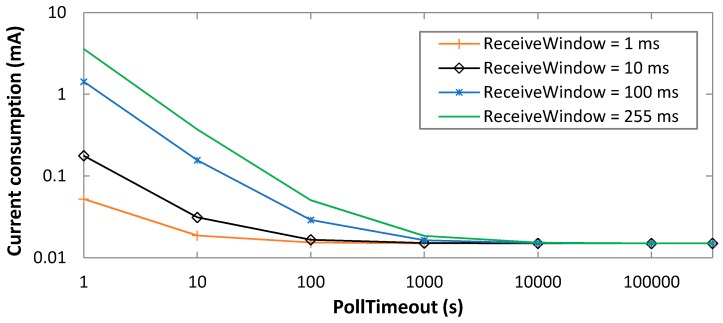
Average current consumption of the BM-LPN, as a function of PollTimeout, in absence of data message transmissions, and for various ReceiveWindow settings.

Firstly, we evaluate the average BM-LPN current consumption, based on Equations (1)–(3), as a function of PollTimeout and ReceiveWindow values that cover the whole allowed range for these parameters (Figure 8). As a benchmark, results in Figure 7 are obtained under the assumption that data messages are not transmitted. 

As shown in Figure 8, the average current consumption decreases with PollTimeout, since for large PollTimeout values, sleep intervals become dominant. The average current consumption increases with ReceiveWindow, since for greater values of this parameter, the BM-LPN radio interface needs to remain in receive mode (thus, consuming a higher amount of current) for a longer time. For example, for PollTimeout = 10 s, the current consumption ranges from 18.7 µA to 371 µA, for ReceiveWindow settings of 1 ms and 255 ms, respectively.

Performance variations for different ReceiveWindow values become irrelevant for a PollTimeout greater than 10,000 s, where sleep intervals have a significantly greater duration than active ones.

**Figure 9:**
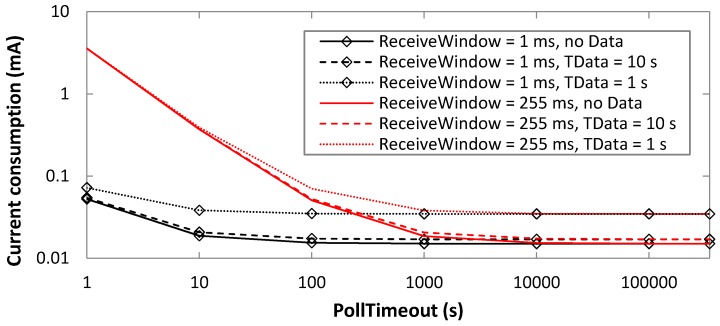
Impact of *T_Data_* on the average current consumption of the BM-LPN, for ReceiveWindow settings of 1 ms and 255 ms, as a function of PollTimeout.

We next study the impact of the time between two consecutive data message transmissions by the sensor, *T_Data_*, on the BM-LPN current consumption. As shown in Figure 9, BM-LPN current consumption decreases with *T_Data_*, since increasing *T_Data_* reduces the rate at which operations related with data transmission are performed. Such increase grows asymptotically with PollTimeout, since with greater values of the latter, data transmission becomes the main activity, other than sleeping, of the BM-LPN. 

A significant current consumption increase can be observed for high data message sending rates such as 1 Hz (i.e., *T_Data_* = 1 s), of a factor up to 2.3 compared with absence of data transmission. However, reducing the data message rate to 0.1 Hz (i.e., *T_Data_* = 10 s) leads to a current consumption only slightly greater than the one obtained in absence of data transmission. 

For high ReceiveWindow settings (e.g., ReceiveWindow = 255 ms), and for low PollTimeout values (e.g., up to 10 s), polling becomes dominant, leading to a high current consumption (2 orders of magnitude greater than the sleep state current consumption) and rendering the impact of *T_Data_* negligible. In that case, data transmission uses the radio interface for a low time, compared with the scan interval duration. Performance differences between the highest and the lowest possible values for ReceiveWindow (i.e., 255 ms and 1 ms, respectively), become negligible for PollTimeout settings beyond 1000 s. In that region of values, the data transmission rate sets a lower bound on current consumption. 

### 5.2. BM-LPN Lifetime 

We next calculate the theoretical lifetime of a battery-operated BM-LPN, for the same range of scenarios considered in the previous subsection, by using Equation (4), and the current consumption results obtained in the previous subsection. We assume an ideal battery with a capacity of 235 mAh (e.g., as featured by the prevalent CR2032 button cell battery), and a self-discharge rate equal to 1%/year of its initial capacity [19].

**Figure 10:**
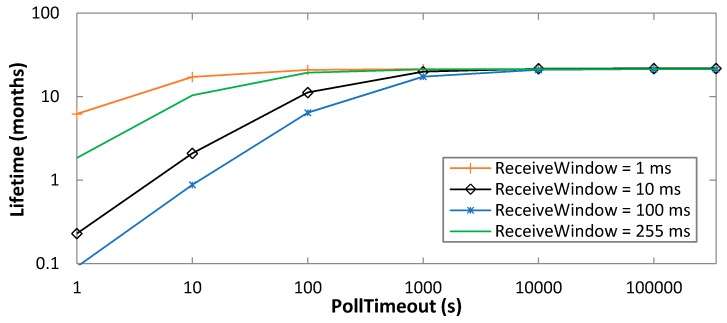
BM-LPN lifetime, as a function of PollTimeout, in absence of data message transmissions, and for different ReceiveWindow settings.

Figure 10 depicts the lifetime of the BM-LPN, in absence of data message transmission, for the same PollTimeout and ReceiveWindow settings considered in Figure 8. The theoretical BM-LPN lifetime is inversely proportional to the average current consumption. BM-LPN lifetime grows with PollTimeout, with an asymptotic lifetime of 21.4 months that is limited by the sleep state current consumption. For low PollTimeout values, the ReceiveWindow setting becomes relevant, since active states have relatively significant duration and current consumption compared to sleep intervals. For example, for PollTimeout = 10 s, the BM-LPN lifetime ranges from 0.87 months to 17.1 months, for ReceiveWindow settings of 255 ms and 1 ms, respectively. However, performance differences decrease with PollTimeout, with BM-LPN lifetime results exhibiting negligible differences for PollTimeout values greater than 10,000 s. 

**Figure 11:**
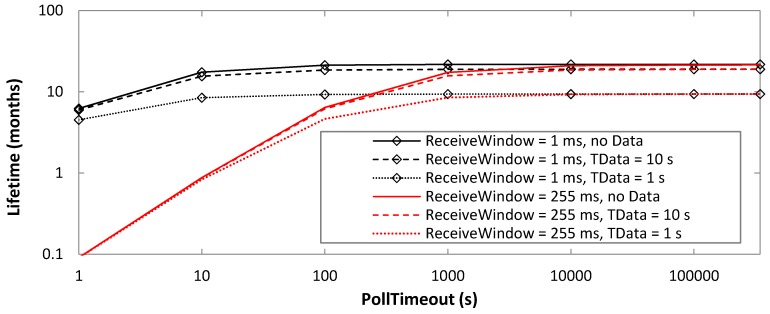
Impact of *T_Data_* on the BM-LPN lifetime, as a function of PollTimeout, for ReceiveWindow values of 1 ms and 255 ms, and as a function of PollTimeout.

As expected, when the battery-operated BM-LPN also transmits data messages, its lifetime decreases (Figure 11). The impact of data transmission is low; for example, there is up to a maximum of 13% lifetime decrease for a relatively high rate of data message transmission such as 0.1 Hz (i.e., *T_Data_* = 10 s). The reason for this is the low contribution of data transmission to current consumption, compared with polling, scanning, and the sleep intervals. Very high data message rates like 1 Hz (i.e., *T_Data_* = 1 s) produce a significant lifetime decrease, by a factor up to 2.3, compared with a BM-LPN that does not transmit data messages.

Impact of data transmission is greater for low ReceiveWindow settings, since in this case current consumption of data message transmission is relevant compared to that of polling and scanning, whereas sleep intervals are not dominant. For example, for PollTimeout = 10 s, setting *T_Data_* to 1 s decreases BM-LPN lifetime by a factor of 2.03 and 1.05, for ReceiveWindow settings of 255 ms and 1 ms, respectively, compared with a lack of data message transmission. 

### 5.3. Energy Consumed per Delivered Bit 

**Figure 12:**
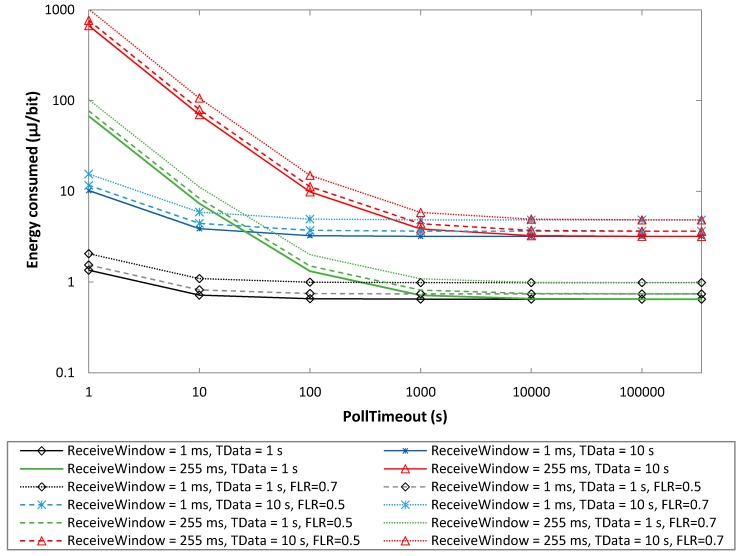
Energy consumed per delivered data bit, as a function of PollTimeout, for different ReceiveWindow, *T_Data_* and FLR values.

In this subsection, we determine the energy consumed per delivered bit, *EC_delivery_*, for a battery-operated BM-LPN, by using Equations (5) and (6). Figure 12 illustrates the results obtained as a function of PollTimeout, for different ReceiveWindow and *T_Data_* settings, and for different FLR values. We assume a data message payload size of 20 bytes. 

For given *T_Data_* and FLR values, *EC_delivery_* exhibits a behavior with PollTimeout that is similar to that of current consumption, that is, an asymptotical decrease with this parameter (see Figure 8). However, *EC_delivery_* increases with *T_Data_*. Note that, within *T_Data_*, the energy consumed during sleep intervals accumulates over a time close to *T_Data_*. Such an increase is greater for low PollTimeout values, where polling and scanning operations become relevant and sleep intervals are shorter. For example, for PollTimeout = 1 s, *EC_delivery_* for *T_Data_* = 10 s is 7.5 times greater than that obtained for *T_Data_* = 1 s, while as PollTimeout increases, the *EC_delivery_* difference between using the same respective *T_Data_* settings tends to a factor of 4.9.

A non-zero FLR increases *EC_delivery_*, since energy is consumed in all messages transmitted, however, a subset of the messages are not delivered. Because a message is transmitted by sending three advertisements, the energy consumed increases significantly only for relatively high FLR values. For example, FLR = 0.5 leads to an *EC_delivery_* increase of 14%. For FLR values beyond 0.5, the *EC_delivery_* increase grows quickly (e.g., FLR = 0.7 yields an *EC_delivery_* increase of 52%). 

## 6. Network Lifetime and Architectural Design of Bluetooth Mesh

In the last two sections, we modeled and evaluated the energy consumption performance parameters of a BM-LPN. Such a node is connected at the edge of the network, via a neighboring BM-FN. The Bluetooth Mesh specification enables the connectivity of a BM-LPN while allowing its low-power operation by keeping the BM-FN always available for receiving possible incoming packets (which may be intended for or originated by the BM-LPN). Similarly, Bluetooth Mesh-Relay Nodes (BM-RNs) are also required to be always ready for receiving and forwarding messages. Therefore, the Bluetooth Mesh architecture is based on a backbone of always-on BM-FNs and BM-RNs, which are by default in receive mode, and thus generally consume a significant amount of current (e.g., 13.9 mA, see Table 2). This approach is suitable for use cases where it is feasible to use the electricity grid as the power source for the backbone, and in fact home automation (one of such use cases) is one of the main target applications of Bluetooth Mesh. However, in many environments, an electricity grid socket is not present near the device that needs to be powered. Batteries do not offer a proper alternative, since using a 2400 mAh battery, a BM-FN or a BM-RN based on the hardware platform considered in this paper will have a lifetime of only 7.2 days. This figure would also limit network lifetime to the same value. Another option would be using powerful enough energy harvesting sources, but this approach may be expensive, and may not always be practical. Therefore, Bluetooth Mesh cannot be considered as a general purpose IoT technology, and its application is limited to the scenarios where its backbone can be appropriately powered.

## 7. Conclusions

In this paper, we have presented analytical models on important energy performance parameters of a battery-operated BM-LPN, such as current consumption, lifetime and energy consumed per delivered bit. The models take into account the influence of Bluetooth Mesh parameters, such as ReceiveWindow and PollTimeout, as well as the data message rate. We also evaluate the influence of losses on the energy consumed per delivered bit. We have assumed a BM-LPN that corresponds to a sensor device that periodically transmits data messages. 

BM-LPN current consumption decreases with PollTimeout, and increases with ReceiveWindow. For high PollTimeout values, using different ReceiveWindow settings leads to negligible performance differences. Remarkably, the data message transmission rate is irrelevant in terms of current consumption, except for very high sending rates in the order of 1 Hz or greater. 

Assuming a 235 mAh battery, the asymptotic lifetime for the considered BM-LPN hardware platform is 21.4 months. For low PollTimeout values, the ReceiveWindow setting becomes relevant, due to the energy consumption of the BM-LPN during active states, compared to that of sleep intervals. Data transmission reduces BM-LPN lifetime to a greater extent for low ReceiveWindow settings, due to the lower impact of sleep intervals.

The energy consumed by the BM-LPN by each delivered bit increases with *T_Data_*, as in fact the energy consumed during sleep intervals is significant. Message losses increase the energy cost of data delivery. However, since data transmission is carried out over 3 advertisements, such an increase is only significant for very high FLR (e.g., the increase is greater than 10% for an FLR beyond 0.46).

For a given Bluetooth Mesh network scenario, a suitable parameter configuration will need to take into account energy performance aspects (e.g., by using the models presented in this paper), as well as specific characteristics of the scenario and the intended application requirements. Note that Bluetooth Mesh cannot be considered as a general purpose IoT technology, since its application is limited to the scenarios where its backbone (i.e., BM-FNs and BM-RNs) can be appropriately powered.

## Figures and Tables

**Table 1:** Characterization of the states related with the transmission of three advertisements by an nRF51422 Development Kit.

State Number	Description	Duration		Current Consumption	
		Variable	Value (ms)	Variable	Value (mA)
0	Sleep	*T_sleep_*	Equation (2)	*I_sleep_*	0.015
1	Wake up	*T_wake-up_*	1.51	*I_wake-up_*	0.38
2	1st transmission	*T_tx_*_1_	0.52	*I_tx_*_1_	8.45
3	2nd transmission	*T_tx_*_2_	0.53	*I_tx_*_2_	8.85
4	3rd transmission	*T_tx_*_3_	0.53	*I_tx_*_3_	8.71
5	1st channel change	*T_ch_change__*_1_	0.30	*I_ch_change__*_1_	3.66
6	2nd channel change	*T_ch_change__*_2_	0.33	*I_ch_change__*_2_	3.91
7	Radio off	*T_radio_off_*	0.29	*I_radio_off_*	5.48
8	Post-processing	*T_post_*	0.62	*I_post_*	1.2
9	Cool down	*I_cool_down_*	14.0	*I_cool_down_*	0.074

**Table 2:** Characterization of the states related with performing a scan interval by an nRF51422 Development Kit.

State Number	Description	Duration		Current Consumption	
		Variable	Value (ms)	Variable	Value (mA)
10	Wake up pre scan	*T_wake-up_scan_*	1.57	*I_wake-up_scan_*	0.34
11	Scan	*T_scan_*	ReceiveWindow	*I_scan_*	13.9
12	Radio off & processing	*T_radio_off_*	0.32	*I_radio_off_*	6.44
13	Cool down	*T_post_*	26.3	*I_post_*	0.07
